# Development of a Colorimetric Loop-Mediated Isothermal Amplification Assay for the Detection of *Trypanosoma cruzi* in Low-Resource Settings

**DOI:** 10.3390/diagnostics14111193

**Published:** 2024-06-05

**Authors:** Taylor J. Moehling, Myla D. Worthington, Pui-Yan G. Wong, Season S. Wong, Robert J. Meagher

**Affiliations:** 1 Department of Biotechnology and Bioengineering, Sandia National Laboratories, Livermore, CA 94551, USA; tjmoehl@sandia.gov (T.J.M.); mywor1@morgan.edu (M.D.W.); 2 AI Biosciences, Inc., College Station, TX 77845, USA

**Keywords:** loop-mediated isothermal amplification, colorimetric output, Chagas disease, *Trypanosoma cruzi*

## Abstract

Chagas disease is an inflammatory parasitic infection caused by *Trypanosoma cruzi* (*T. cruzi*). Early diagnosis is crucial in guiding treatment and slowing disease progression; however, current diagnostic methods have insufficient detection limits and often require skilled technicians. Molecular tests, especially isothermal nucleic acid assays, are advantageous due to their excellent sensitivity, specificity, speed, and simplicity. Here, we optimized a colorimetric loop-mediated isothermal amplification (LAMP) assay for *T. cruzi*. We can detect as few as 2 genomic copies/reaction using three different *T. cruzi* strains. We examined selectivity using other parasitic protozoans and successfully detected *T. cruzi* DNA extracted from parasites in human whole blood down to 1.2 parasite equivalents/reaction. We also performed a blinded study using canine blood samples and established a 100% sensitivity, specificity, and accuracy for the colorimetric LAMP assay. Finally, we used a heated 3D printer bed and an insulated thermos cup to demonstrate that the LAMP incubation step could be performed with accessible, low-cost materials. Altogether, we have developed a high-performing assay for *T. cruzi* with a simple colorimetric output that would be ideal for rapid, low-cost screening at the point of use.

## 1. Introduction

Chagas disease is a neglected tropical illness caused by the parasite *Trypanosoma cruzi* (*T. cruzi*) that affects 6–8 million people globally [1]. *T. cruzi* is organized into six discrete typing units, with TcI being the most prevalent overall (~30% of human infections) and TcVI being associated with domestic transmission cycles [2]. Transmission routes for Chagas disease include direct contact with the feces of an infected triatomine bug, congenital transfer from mother to child, exposure to contaminated blood products, and consumption of contaminated food or beverages [1]. For many years, Chagas was considered a rural disease; however, climate change and urbanization have made it a widespread public health problem [3]. Infection can be either acute or chronic, with symptoms ranging from fever and swelling to heart failure and enlarged organs. Chagas disease is most prevalent in Latin America, though there are now more than 300,000 people living with the disease in the United States. If left untreated, Chagas disease can be life-threatening, with approximately 12,000 deaths reported annually worldwide [4].

In recent years, there have been an increasing number of efforts to identify novel *Trypanosoma* biomarkers and improve detection techniques [5]. Early diagnosis during the acute disease stage is critical because it allows prompt pharmacological treatment, thereby reducing morbidity and mortality [6]. Acute Chagas disease is typically diagnosed through microscopic analysis of blood smears or polymerase chain reaction (PCR). Chronic infections typically require serological testing, but reactivation can be confirmed via PCR [7,8]. Blood smears and serology tests both have highly variable sensitivities and specificities. While PCR has excellent performance metrics, it requires expensive equipment for thermal cycling and results often take ≥2 hours to obtain. Loop-mediated isothermal amplification (LAMP) is a PCR alternative that is faster and requires minimal equipment [9]. Researchers have developed LAMP assays to detect *T. cruzi* and have demonstrated improvements in assay sensitivity, speed, and portability compared to PCR. However, some of these LAMP assays rely on instrumentation for signal detection, which increases the overall cost [10,11]. Others have had success using colorimetric indicators such as SYBR Green, but the dye inhibits LAMP amplification, meaning it must be added post-amplification, significantly increasing the likelihood of carryover contamination [12,13,14]. Other dyes that are compatible with LAMP have also been tested, but many result in subtle color changes, which leaves room for user error [15,16,17]. In this work, we optimized a closed-tube LAMP assay for *T. cruzi* that produces an obvious color shift (pink to yellow) that can be interpreted by the naked eye. We also demonstrated automated sample preparation with an in-house device and efficient assay heating with low-cost tools. Altogether, this LAMP test would enable rapid, inexpensive Chagas screening in low-resource settings. 

## 2. Materials and Methods

### 2.1. Loop-Mediated Isothermal Amplification (LAMP)

LAMP reactions were 10 µL total volume: 1.0X WarmStart colorimetric LAMP master mix (NEB), 2 µM Syto9 (Invitrogen, Carlsbad, CA, USA), 20 mM Guanidine Hydrochloride (Sigma-Aldrich, Burlington, MA, USA), 0.2 µM of F3 and B3 primers, 1.6 µM of FIP and BIP primers, 0.8 µM of LF and LB primers (IDT), and 2 µL of template. Primer sequences were obtained from Ordonez et al. (Table S1) and targeted the highly repetitive nuclear satellite region of *T. cruzi* [12]. 

### 2.2. Limit of Detection (LOD), Selectivity, and Contrived Clinical Sample Experimental Design

We used three strains of purified DNA for limit-of-detection (LOD) analysis: Dm28c (TcI), G (TcI), and CL (TcVI) (BEI Resources, Manassas, VA, USA). The DNA was tested at 100, 50, 20, 10, 5, and 2 genome copies/reaction. The no template controls (NTC) contained nuclease-free water in place of template. We included 8 replicates at each concentration and 16 NTC replicates. LAMP was run for 30 min at 65 °C on a pre-heated CFX96 Real-Time PCR Detection System block (Bio-Rad, Hercules, CA, USA). For selectivity testing, we used *Cryptosporidium parvum* (ATCC, Manassas, VA, USA), *Leishmania infantum* strain MHOM/TN/80/IPT-1 (ATCC), and *Plasmodium falciparum* strain 3D7 (ATCC) at 500 genome copies/reaction. For contrived clinical sample testing, *T. cruzi* cells (TcI) were spiked in commercially sourced human whole blood (Innovative Research, Novi, MI, USA) using a stock solution at 6000 parasite equivalents/µL (1 parasite contains 1 copy of genomic DNA). The samples were further diluted in *T. cruzi* negative human whole blood to concentrations of 60, 6, 0.6, 0.06, and 0.006 parasite equivalents/µL. Magnetic bead-based DNA extraction was performed using a previously designed sample preparation device [18,19] combined with one of two kits: (1) MagMAX DNA Ultra 2.0 Kit (ThermoFisher, Waltham, MA, USA) or (2) NucliSENS easyMAG Kit (bioMerieux, Marcy-l’Étoile, France) with mineral oil and 70% isopropyl alcohol replacing wash buffers. The reaction conditions remained the same, except that samples were incubated for 45 min.

### 2.3. Gold-Standard Comparison Experimental Design

We performed a study to compare gold-standard quantitative PCR (qPCR) to LAMP to determine the sensitivity, specificity, and accuracy of our assay. We used a single-blinded study design to mimic a diagnostic setting; the individual performing LAMP did not know the qPCR test results for any of the samples. We received 20 remnant canine blood samples from a colleague who had previously collected the samples for another study [20]. The canine blood samples were stored in either EDTA or clotting tubes. The blood samples in EDTA were centrifuged to separate plasma, buffy coat layer, and packed red blood cells. DNA extraction was performed on the buffy coat layer (samples 1–13) and clotted blood (samples 14–20) using the NucliSENS easyMAG Kit with mineral oil and 70% isopropyl alcohol replacing wash buffers. qPCR reactions were 20 µL total volume: 1.0X TaqPath One-Step Multiplex Master Mix (ThermoFisher, Waltham, MA, USA), 0.5 μM of forward primer, 0.5 μM of reverse primer, 0.25 μM of FAM probe (LGC Biosearch Technologies, Hoddesdon, UK), and 4 µL of DNA template. Primers were borrowed from Duffy et al. (Table S2) and targeted the highly repetitive nuclear satellite region of *T. cruzi* [21]. The authors reported a qPCR LOD of 0.0007 parasite equivalents/µL. qPCR was run for 2 min at 25 °C, 10 min at 95 °C, then 50 cycles of 15 s at 95 °C followed by 1 min at 58 °C on a CFX96 Real-Time PCR Detection System (Bio-Rad, Hercules, CA, USA). If a cycle threshold (Ct) value was recorded using the auto-threshold setting on the real-time system, the sample was deemed positive. If no Ct value was generated, the sample was considered negative. The same canine blood samples were then tested with our LAMP assay; the reaction conditions remained the same as above and the samples were incubated for 45 min. We ran 8 replicates of each sample and used end-point colorimetric results to determine amplification output. 

### 2.4. Low-Cost Heating Experimental Design

While we optimized the colorimetric *T. cruzi* LAMP assay using a real-time thermal cycler, we also demonstrated incubation with two low-cost tools: a 65 °C heated 3D printer bed (~$180, Monoprice IIIP, Rancho Cucamonga, CA, USA) and a stainless-steel insulated thermos (~$25, Thermos, Schaumburg, IL, USA) filled with 65 °C water. Reactions heated on the 3D printer bed were 25 µL total and DNA template (ATCC) was added so that final concentrations were 4000, 400, 40, and 0 genome copies/reaction. The tubes were placed directly on the heater or in an aluminum block with custom tube slots which was also placed on the heater. Photos of the tubes were taken at 0, 10, 15, 20, and 25 min. For the 25 µL thermos reactions, DNA extracted from *T. cruzi* positive whole blood using the NucliSENS easyMAG Kit (with mineral oil and 70% isopropyl alcohol replacing wash buffers) was added to give final concentrations of 155, 15.5, 1.55, 0.155, 0.0155, and 0 parasite equivalents/reaction. A positive (ATCC, 228 parasite equivalents/reaction) and negative (NTC, water) control were also included. We have previously demonstrated molecular assay incubation using this 24 oz. thermos cup [22]. Simply, a thin, round sponge was trimmed to fit just inside the opening of the thermos and two rows of four holes each were cut for the tubes. The thermos was filled with 65 °C water and the tubes were inserted into the sponge so that they penetrated the thickness and were submerged in the water. The thermos lid was then capped and after 30 min the tubes were removed and imaged.

### 2.5. Statistical Analysis

For the LOD, selectivity, and contrived clinical sample studies, end-point colorimetric results were quantified using RGB analysis in ImageJ v1.54. RGB values were converted to hue, which is often depicted on a color wheel with a range from 0° to 360° [23]. Hue was graphed using quartile box-and-whisker plots in GraphPad Prism 9. To determine significance, a one-way ANOVA with post-hoc Dunnett’s test was performed with multiple comparisons against the NTC or *T. cruzi* positive control with a 95% confidence interval. For the gold-standard comparison study, a 2 × 2 contingency table showing true positive (TP), true negative (TN), false positive (FP), and false negative (FN) values was created to directly compare qPCR and LAMP and to calculate the sensitivity ((TP)/(TP + FP)), specificity ((TN)/(TN + FP)), and accuracy ((TP + TN)/(TP + FP + FN + TN)) of the colorimetric LAMP assay [24]. We also calculated Cohen’s Kappa Statistic (κ) to measure the level of agreement between the two analysis methods for the canine blood samples (qPCR vs. LAMP).

## 3. Results

### 3.1. LAMP Assay Optimization

**Figure 1:**
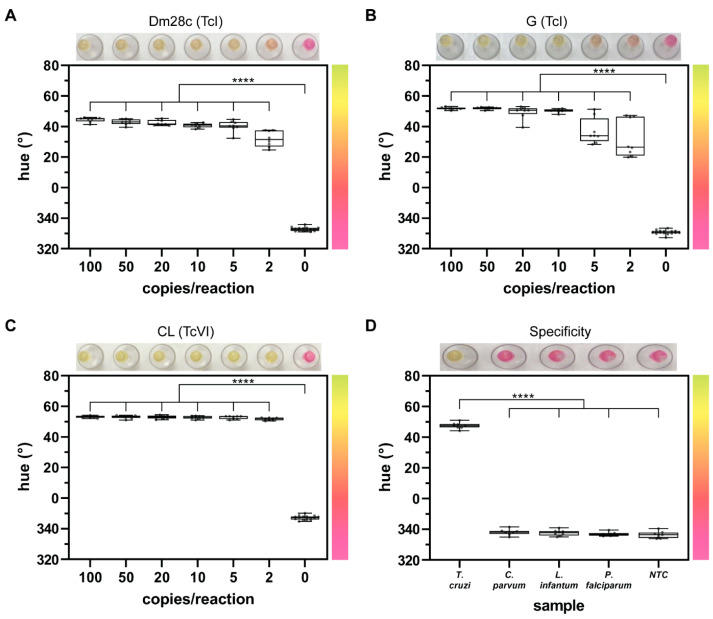
*T. cruzi* LAMP assay LOD and selectivity. Representative end-point colorimetric results (top), graphic demonstrating relationship between color (right) and hue value (y-axis), and quantified hue values (center) are shown for each data set. On a color wheel, a yellow hue (positive) is assigned 45–90° while pink (negative) is 315–360°. (**A**) Hue values for strain Dm28c are statistically significant down to 2 copies/reaction when compared to NTC (0). (**B**) Measured hue values for strain G show a statistically significant difference down to 2 copies/reaction when compared to NTC. (**C**) Hue values for strain CL are statistically significant down to 2 copies/reaction when compared to NTC. (**D**) Measured hue values for *C. parvum*, *L. infantum*, *P. falciparum*, and NTC show a statistically significant difference when compared to *T. cruzi*. *n* = 8 each circle represents a replicate; **** indicates *p* ≤ 0.0001.

The commercial colorimetric LAMP master mix contains phenol red which changes from pink to yellow as the reaction pH drops during amplification. The color difference between positive and negative samples is demonstrated in Figure 1 and can be interpreted by the naked eye. However, we used an objective quantitative analysis based on hue to further examine results. Hue provides a singular numerical value for each sample that closely matches what the human eye perceives [23,25]. On a color wheel, a yellow hue (positive) is assigned 45–90° while pink (negative) is 315–360°.

During the assay design and optimization phase, we modified the Ordonez et al. primers so that all six fit within a single repeat of the nuclear satellite gene. Interestingly, this seemingly minute change reduced assay performance, so we reverted to the original primer set. We also determined that pre-heating the block prior to sample addition decreased the incidence of false positives. Exposing enzymes and primers to sub-optimal temperatures during the heating ramp-up could result in primer-dimer extension artifacts, especially at temperatures above the activation point for Bst WarmStart DNA Polymerase (45 °C) but below the final reaction temperature (65 °C). 

### 3.2. Assay LOD and Selectivity 

For LOD experiments, we used Dm28c, G, and CL strains of *T. cruzi* and added 2–100 genome copies/reaction. We saw obvious differences between positive and negative samples at end point (Figure 1A–C and Figure S1–S3). Furthermore, there was a statistically significant difference in the hue values from 2–100 copies/reaction when compared to the NTC (0) for all three strains tested (Figure 1A–C, *p* ≤ 0.0001). This LOD of 2 copies/reaction, or 1 copy/µL, is comparable to other LAMP assays detecting *T. cruzi* [12].

Many researchers have reported high rates of *T. cruzi* primer cross-reactivity, so it is important to confirm assay selectivity by testing several other parasitic protozoans [7]. There was no amplification, and therefore no color change, of samples containing *C. parvum, L. infantum*, or *P. falciparum* (Figure 1D and Figure S4). There was a statistically significant difference in the colorimetric output of the *C. parvum*, *L. infantum*, *P. falciparum*, and NTC samples when compared to *T. cruzi* (*p* ≤ 0.0001) and the off-target parasites resulted in hue values that were indistinguishable from NTC samples (*p* > 0.2). Together, this illustrates that other parasitic protozoans that also cause human disease will not interfere with assay results.

### 3.3. Contrived Clinical Sample Testing

**Figure 2:**
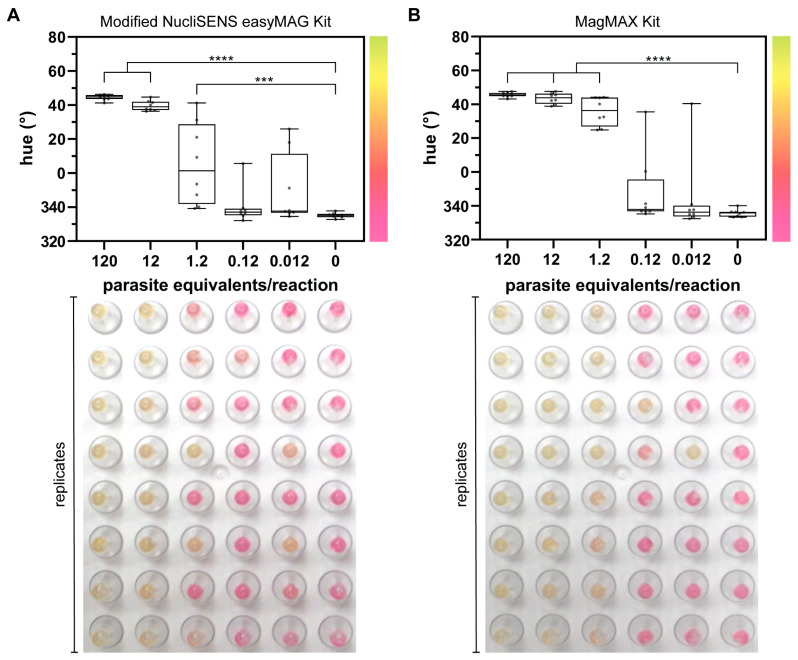
Detection of *T. cruzi* DNA extracted from parasites in human whole blood. Highlighted for each data set are quantified hue values (center), graphic demonstrating relationship between color (right) and hue value (y-axis), and end-point colorimetric results (bottom). Each column in the 96-well plate is aligned with the corresponding concentration on the x-axis of the graph above. (**A**) DNA extraction was performed using the modified NucliSENS easyMAG Kit. There is a statistically significant difference in the hue value of LAMP products from as few as 1.2 parasite equivalents/reaction as compared to the negative control (0). (**B**) DNA extraction was conducted with the MagMAX Kit. There is a statistically significant difference in hue value down to 1.2 parasite equivalents/reaction when compared to NTC. *n* = 8 each circle represents a replicate; *** indicates *p* ≤ 0.001; **** indicates *p* ≤ 0.0001.

To determine whether this colorimetric LAMP assay is compatible with contrived clinical samples, we spiked parasites in human whole blood and extracted the DNA using two different methods. We explored two extraction protocols to see whether the performance of the downstream colorimetric LAMP assay changed with the extraction technique. As seen in the bottom panels in Figure 2, end-point colorimetric results for the contrived clinical samples were more variable between replicates compared to purified genomic DNA. Still, statistical analysis demonstrated significance down to 1.2 parasite equivalents/reaction, or 0.6 parasite equivalents/µL, for both extraction methods (Figure 2A,B, Figures S5 and S6, *p* ≤ 0.001). Other groups illustrated similar findings when comparing results from purified nucleic acids and mock clinical samples [13]. From these experiments, we found that samples extracted with the unmodified MagMAX Kit more consistently amplify at middle-range concentrations (Figure 2B) but both methods provide highly purified DNA that is necessary for subsequent amplification. We note that we replaced the wash buffers of the NucliSENS easyMAG Kit with mineral oil and 70% isopropyl alcohol to illustrate that cheaper reagents that can be sourced from drug stores and pharmacies can be successfully used for DNA extraction.

### 3.4. Direct Comparison of LAMP and PCR Performance with Canine Samples

To determine the sensitivity, specificity, and accuracy of the *T. cruzi* LAMP assay, we directly compared it to qPCR. We believe this is a valid comparison because primers for both assays target the same nuclear satellite region, meaning the calculated copy number for both assays should be equivalent. The first researcher performed qPCR on all 20 of the extracted canine samples and recorded the Ct values that can be seen in Table 1. The same samples were then handed off to the second researcher, who was blinded to the qPCR results, for analysis via LAMP. There were four samples (4, 8, 9, 11) that were qPCR negative, in which we observed a color change in 1–2 of the LAMP replicates. We did not notice any false positives during assay characterization or contrived sample testing, only during this study with canine blood samples. In the future, it would be worth investigating whether these are indeed false positives or if the LAMP assay is sometimes identifying ultra-low concentration samples that were undetectable via qPCR. We ultimately decided that samples with an end-point color shift in at least half of the replicates (4/8) would be considered positive by LAMP. Using this cutoff, we determined that six samples were positive when amplified via LAMP, while fourteen were considered negative (Table 1, Figures S7 and S8). To directly compare the LAMP assay with gold-standard qPCR, we constructed a 2 × 2 contingency table, seen in Table 2. Overall, there were six true positives, zero false positives, zero false negatives, and fourteen true negatives. From these values, we calculated the sensitivity, specificity, and accuracy all to be 100% for the LAMP assay [26]. Even though the sample size used in this blinded study is fairly small, the data showcase excellent alignment between qPCR and LAMP (κ = 1.0), demonstrating the diagnostic promise of this LAMP assay. This characterization of our LAMP assay demonstrates its ability to sensitively, specifically, and accurately detect trace amounts of *T. cruzi* in clinical samples. 

### 3.5. LAMP Incubation with Inexpensive Heating Elements 

**Figure 3:**
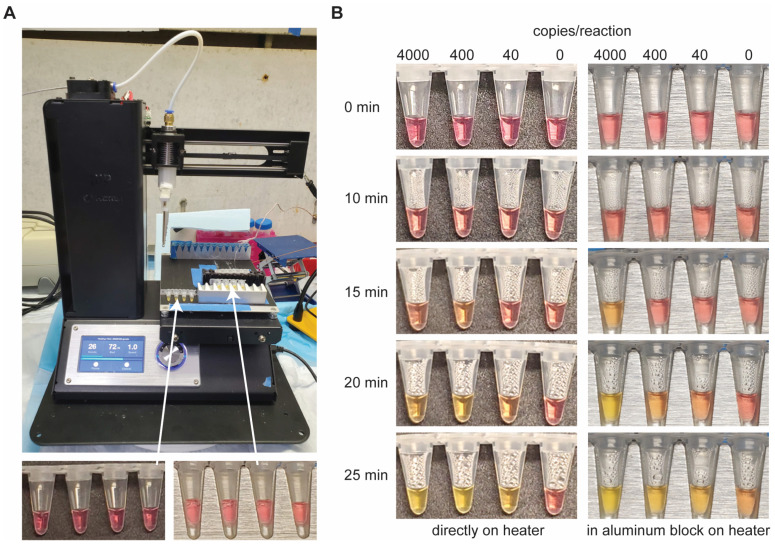
LAMP heating using a low-cost 3D printer (Monoprice IIIP). (**A**) 3D printer setup for LAMP incubation. Samples were either placed directly on the heater or in an aluminum block that was on the heated bed. (**B**) Photos were taken at different time intervals to show the color change over time in positive samples.

Next, we wanted to show that this LAMP assay can be performed with non-traditional laboratory equipment. We first used the heated bed of a low-cost 3D printer to incubate the reactions (Figure 3A). Half the samples were placed directly on the heater while the others were added to the custom aluminum block which was positioned on the heater. Prior to any heating, all samples were pink (Figure 3B, top row). Although the aluminum block provided better contact and heat transfer to the solution, it took longer to reach reaction temperature and the temperature was more difficult to maintain due to added mass. There was no change in color after 10 min of incubation (Figure 3B), but at 15 min, the highest concentration sample in the aluminum block had turned orange (Figure 3B, right). At 20 min, tubes with 4000 copies turned yellow and samples with 400 and 40 copies began to shift to orange (Figure 3B). By the end of the reaction (25 min), all positive samples were yellow in color, while NTC samples remained pink (Figure 3B, bottom row). We did not test concentrations lower than 40 copies/reaction as our initial goal was to demonstrate the feasibility of a simple mechanism to sustain reaction temperature. We noticed condensation in samples placed directly on the heater (Figure 3B, bottom row, left), likely due to poor contact with the heated surface, but this did not seem to affect assay performance. 

**Figure 4:**
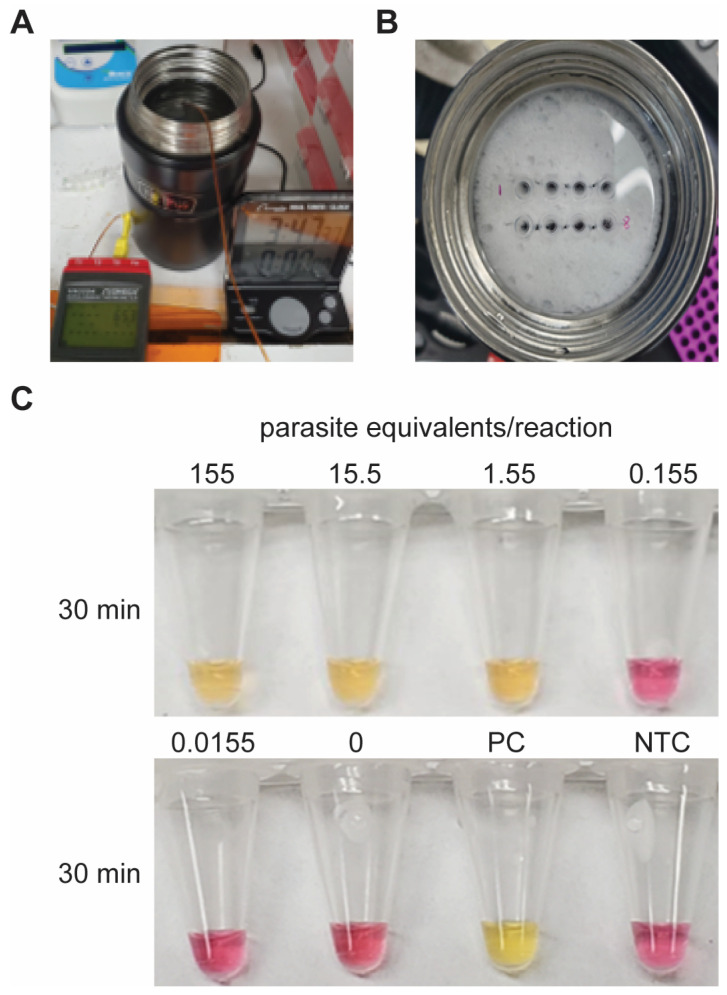
Incubation of LAMP reactions using an inexpensive thermos cup. (**A**) The thermos filled with 65 °C water. (**B**) Sample tubes were loaded into the sponge so they were in contact with the hot water and afterwards, the thermos lid was sealed to prevent evaporation. (**C**) End-point image of the reactions. Samples with no template or low concentrations of target DNA remained pink while the positive control (PC) and samples with higher amounts of template turned yellow.

In the second experiment, we incubated LAMP reactions in an insulated stainless steel thermos cup filled with 65 °C water (Figure 4A,B). After 30 min of heating, the three samples with the highest concentration of template (155, 15.5, and 1.55 parasite equivalents/reaction), along with the positive control (PC), had turned yellow (Figure 4C). No color change was observed for lower concentrations (0.155 and 0.0155 parasite equivalents/reaction) or the negative control (NTC) (Figure 4C). It is worth noting that the detection limit using the thermos for heating is similar to that in Figure 2 in which samples were incubated with a thermal cycler. The last two experiments illustrate that this colorimetric *T. cruzi* LAMP assay is sensitive, but also that it can be conducted with low-cost, simple heating tools, making it ideal for point-of-use testing.

## 4. Discussion

Here, we demonstrate a colorimetric LAMP assay for *T. cruzi* that can detect as few as 2 genomic copies/reaction in just 30 minutes. The colorimetric LAMP test selectively identified *T. cruzi* while off-target parasites such as *C. parvum*, *L. infantum*, and *P. falciparum* did not interfere with amplification or detection. When *T. cruzi* parasites were spiked into human whole blood and DNA was subsequently extracted using a custom, low-cost automated sample preparation device, we were able to reliably detect 1.2 parasite equivalents/reaction using our colorimetric LAMP assay. This is on the same order as other reported dye-based LAMP assays [13,16]. We directly compared colorimetric LAMP to qPCR in a single-blinded study using canine blood samples and calculated an overall sensitivity, specificity, and accuracy of 100% for LAMP. Finally, we demonstrated that LAMP incubation could be achieved with low-cost, accessible items such as a 3D printer or a thermos cup.

In addition to its excellent performance, our assay has a colorimetric output that is easily interpreted by the naked eye. Distinguishing a negative (pink) from a positive (yellow) sample is straightforward, especially compared to other colorimetric LAMP assays where the color shift is quite subtle [15,16,17]. Moreover, phenol red dye does not interfere with amplification and can be incorporated directly into the reaction mixture. This is an improvement over recent publications in which the dye is inhibitory, and the tube must be opened post-amplification [12,13,14]. It is worth noting that pH-based LAMP can be challenging when working with large sample volumes that could affect the overall reaction pH and lead to inaccurate results. In this work, the template volume never exceeded 20% of the total volume and dilutions were made in water or Tris buffer at pH 8. In the future, we could combine the automated sample preparation device and a simple heating unit into a single platform to streamline the testing process and eliminate hands-on steps, leading to an inexpensive Chagas screening tool for low-resource settings. Altogether, we have demonstrated the accuracy, simplicity, and robustness of this colorimetric LAMP assay for detection of *T. cruzi* in blood samples and its compatibility with point-of-use testing.

## Figures and Tables

**Table 1:** Results from the blinded study with canine blood samples. qPCR was used to generate Ct values and determine whether each sample was positive or negative. Sample positivity for the 8 replicates tested via LAMP was determined by end-point colorimetric output.

Sample	qPCR Ct	LAMP (Positive Replicates)
1	--	0/8
2	--	0/8
3	--	0/8
4	--	2/8
5	--	0/8
6	33.81	8/8
7	--	0/8
8	--	1/8
9	--	1/8
10	--	0/8
11	--	1/8
12	--	0/8
13	--	0/8
14	35.19	8/8
15	34.59	7/8
16	34.25	7/8
17	31.61	8/8
18	33.36	8/8
19	--	0/8
20	--	0/8

**Table 2:** 2 × 2 contingency table used to calculate sensitivity, specificity, and accuracy.

		qPCR	
		Positive	Negative
**LAMP**	**Positive**	TP = 6	FP = 0
	**Negative**	FN = 0	TN = 14
		P = 6	N = 14

## Data Availability

The raw data supporting the conclusions of this article will be made available by the authors on request.

## Supplementary Materials

The following supporting information can be downloaded at: https://www.mdpi.com/article/10.3390/diagnostics14111193/s1, Table S1: LAMP primers targeting the nuclear satellite region of *T. cruzi*; Table S2: qPCR primers and probe targeting the nuclear satellite region of *T. cruzi*; Figure S1: *T. cruzi* LAMP assay LOD using strain Dm28c (TcI); Figure S2: *T. cruzi* LAMP assay LOD using strain G (TcI); Figure S3: *T. cruzi* LAMP assay LOD using strain CL (TcVI); Figure S4: *T. cruzi* LAMP assay selectivity; Figure S5: Detection of *T. cruzi* DNA extracted with modified NucliSENS easyMAG Kit; Figure S6: Detection of *T. cruzi* DNA extracted with MagMAX Kit; Figure S7: Identification of *T. cruzi* from canine blood samples—group 1; Figure S8: Identification of *T. cruzi* from canine blood samples—group 2.
