# Supplementary material for: Development of a Colorimetric Loop-Mediated Isothermal Amplification Assay for the Detection of Trypanosoma cruzi in Low-Resource Settings
Source: Diagnostics (Basel). 2024 Jun 5;14(11):1193. doi: 10.3390/diagnostics14111193 (PMC11172009; doi:10.3390/diagnostics14111193)
Supplement: Supplementary file 1 [file diagnostics-14-01193-s001.zip › diagnostics-2965449-supplementary.pdf]

Rapid, Colorimetric Detection of *Trypanosoma cruzi* via Loop-Mediated Isothermal Amplification

Taylor J. Moehling[1], Myla D. Worthington[1], Pui-Yan G. Wong[2], Season S. Wong[2,*], Robert J. Meagher[1,*]

[1] Department of Biotechnology and Bioengineering, Sandia National Laboratories, Livermore, CA, USA
[2] AI Biosciences, Inc., College Station, TX, USA
[*] Correspondence: season.wong@aibiosciences.com; rmeaghe@sandia.gov

Supplementary Data

Table S1: LAMP primers targeting the nuclear satellite region of *T. cruzi*..........................2

Table S2: qPCR primers and probe targeting the nuclear satellite region of *T. cruzi*.........2

Figure S1: *T. cruzi* LAMP assay LOD using strain Dm28c (TcI)..3

Figure S2: *T. cruzi* LAMP assay LOD using strain G (TcI)..3

Figure S3: *T. cruzi* LAMP assay LOD using strain CL (TcVI)...4

Figure S4: *T. cruzi* LAMP assay selectivity...4

Figure S5: Detection of *T. cruzi* DNA extracted with modified NucliSENS easyMAG Kit....5

Figure S6: Detection of *T. cruzi* DNA extracted with MagMAX Kit....................................5

Figure S7: Identification of *T. cruzi* from canine blood samples – group 1.......................6

Figure S8: Identification of *T. cruzi* from canine blood samples – group 2.......................7

Table S1: LAMP primers targeting the nuclear satellite region of *T. cruzi*. Borrowed from Ordonez *et al*.

**Primer**	**Sequence (5' to 3')**
F3	AACTATCCGCTGCTTGGA
B3	AAGAGCTCGCGAAATTCC
FIP	CCCACCATTCACAATCGGAAACCACTCGGCTGATCGTTTT
BIP	AGTCAGAGGCACTCTCTGTCAACCAAGCAGCGGATAGTTC
LF	TTGGACCACAACGTGTGAT
LB	TTCACACACTGGACACCAAA

Table S2: qPCR primers and probe targeting the nuclear satellite region of *T. cruzi*. Borrowed from Duffy *et al*.

**Primer**	**Sequence (5' to 3')**
Forward	ACTCGGCTGATCGTTTTCGA
Backward	AATTCCTCCAAGCAGCGGATA
Probe	FAM-CACACACTGGACACCAA-NFQ-MGB

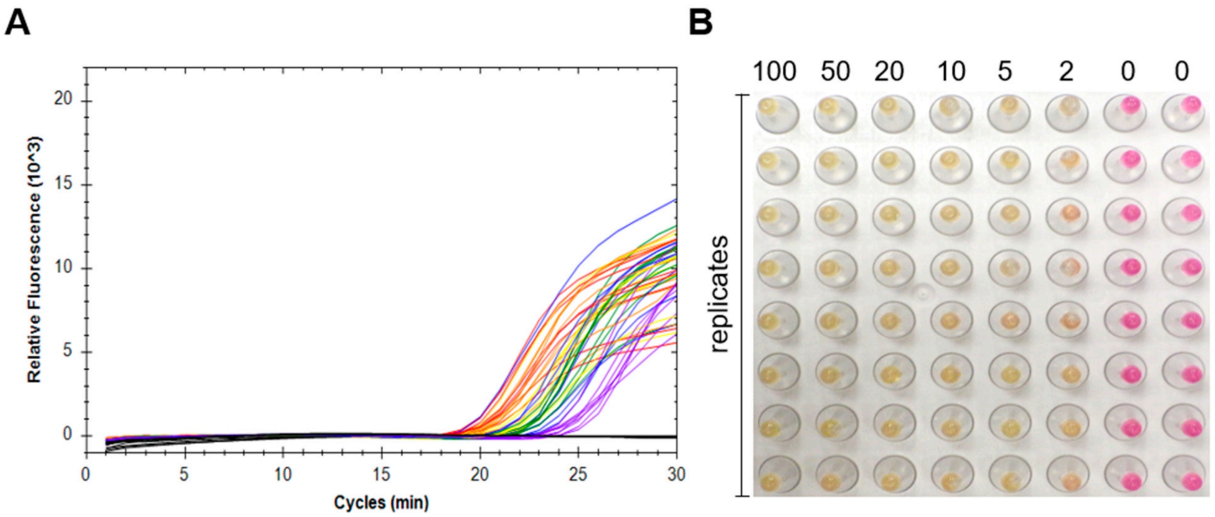

Figure S1: *T. cruzi* LAMP assay LOD using strain Dm28c (TcI). (A) Real-time amplification curves and (B) end-point colorimetric results with 8 replicates at each concentration (16 for negative control). Concentrations tested were 100 (red), 50 (orange), 20 (yellow), 10 (green), 5 (blue), 2 (purple), and 0 (black) genome copies/reaction.

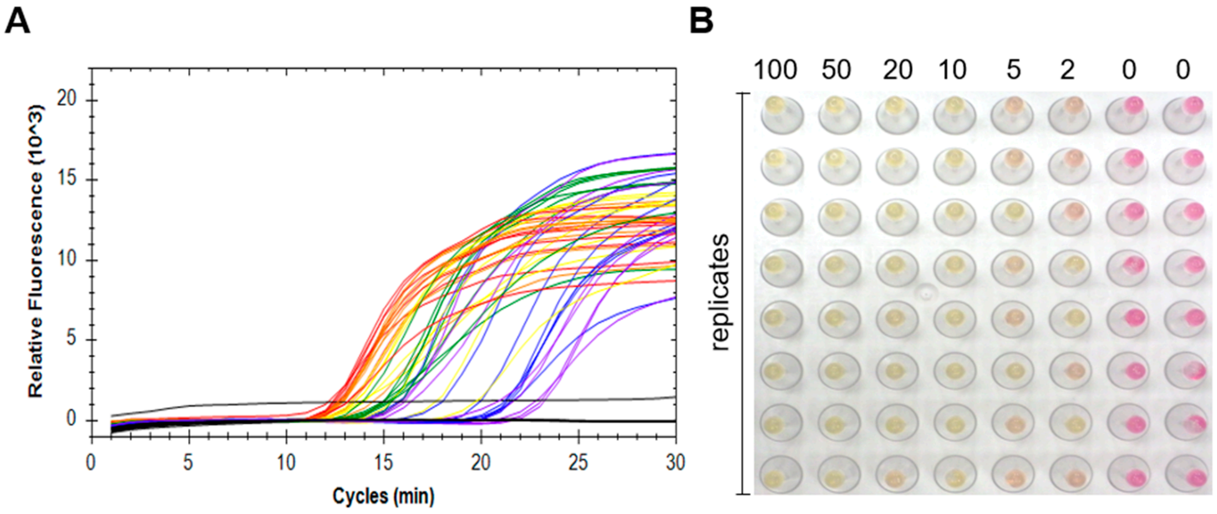

Figure S2: *T. cruzi* LAMP assay LOD using strain G (TcI). (A) Real-time amplification curves and (B) end-point colorimetric results with 8 replicates at each concentration (16 for negative control). Concentrations tested were 100 (red), 50 (orange), 20 (yellow), 10 (green), 5 (blue), 2 (purple), and 0 (black) genome copies/reaction.

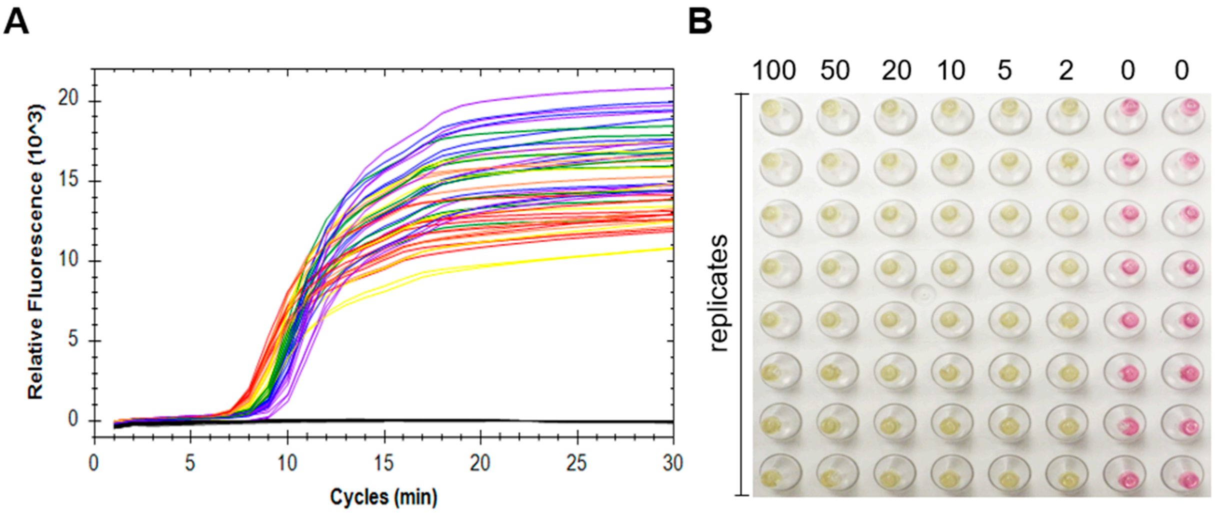

Figure S3: *T. cruzi* LAMP assay LOD using strain CL (TcVI). (A) Real-time amplification curves and (B) end-point colorimetric results with 8 replicates at each concentration (16 for negative control). Concentrations tested were 100 (red), 50 (orange), 20 (yellow), 10 (green), 5 (blue), 2 (purple), and 0 (black) genome copies/reaction.

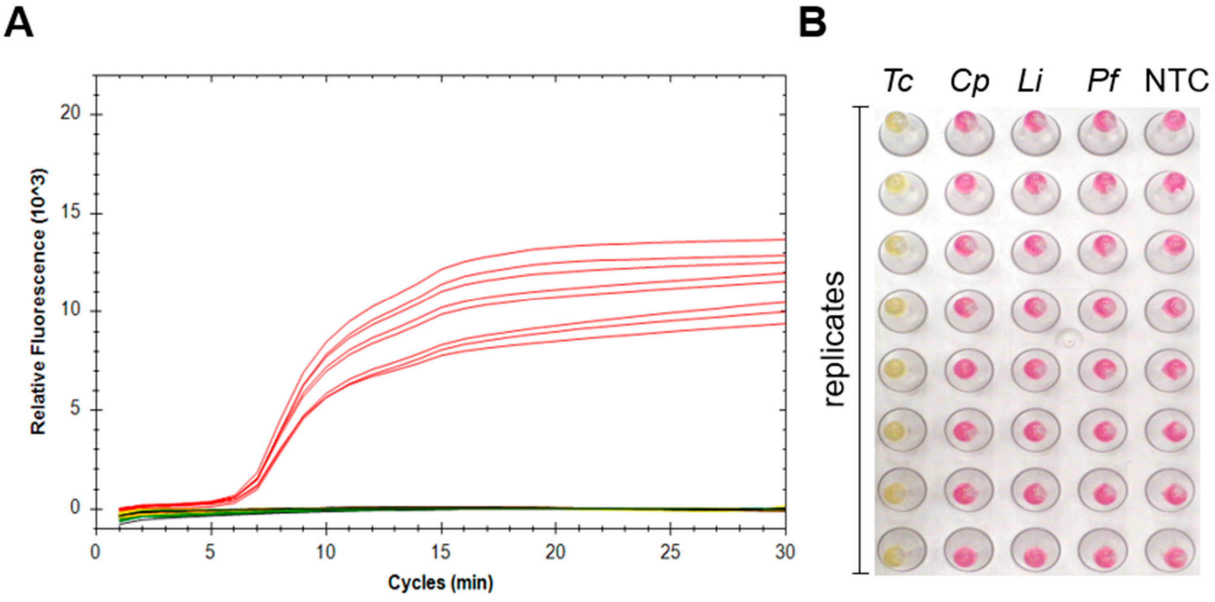

Figure S4: *T. cruzi* LAMP assay selectivity. (A) Real-time amplification curves and (B) end-point colorimetric results with 8 replicates for each sample. *T. cruzi* (red) samples amplified in real time and showed a clear color change at end point in comparison to *C. parvum* (orange), *L. infantum* (yellow), *P. falciparum* (green), and NTC (black) samples. All samples were tested at 500 genome copies/reaction.

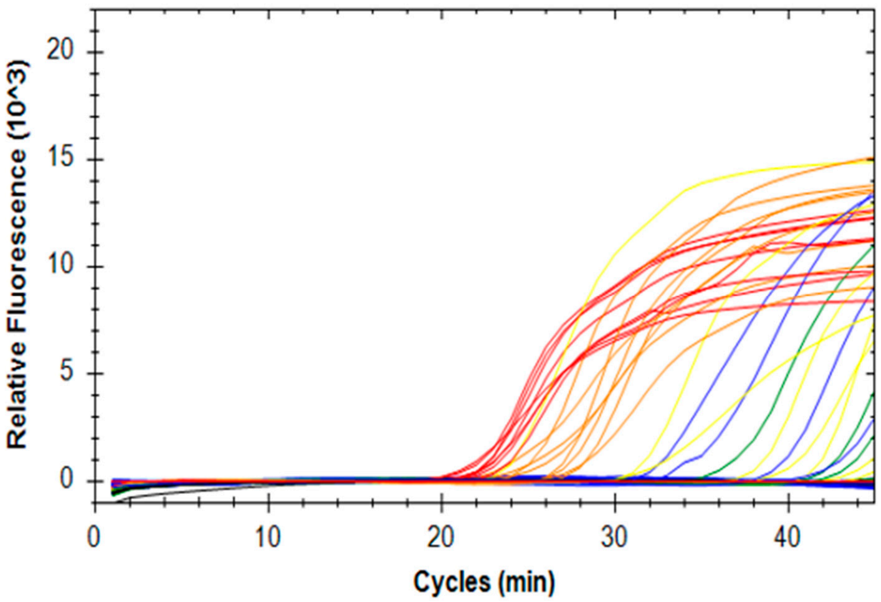

Figure S5: Detection of *T. cruzi* DNA extracted with modified NucliSENS easyMAG Kit. *T. cruzi* parasites were spiked into human whole blood prior to extraction and then used at 120 (red), 12 (orange), 1.2 (yellow), 0.12 (green), 0.012 (blue), and 0 (black) parasite equivalents/reaction. Real-time curves show amplification at higher concentrations.

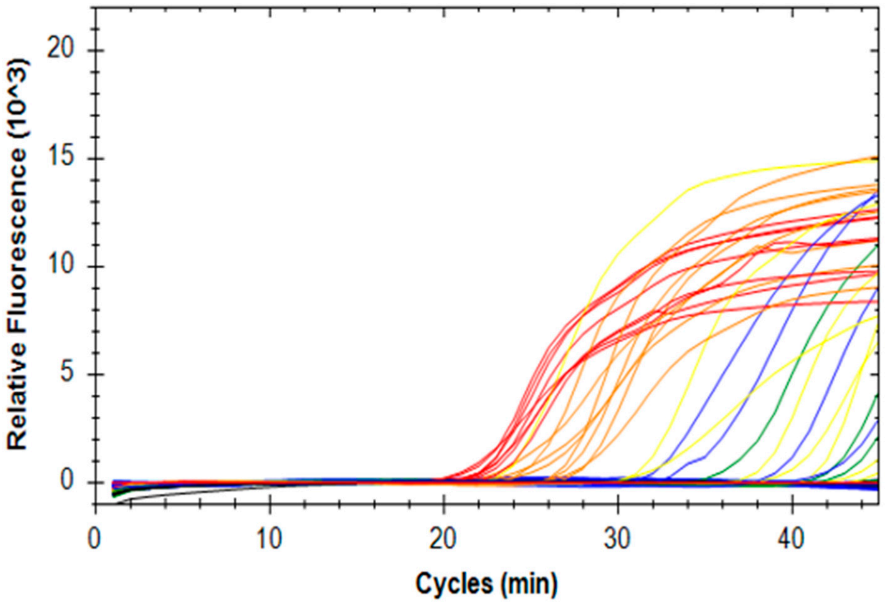

Figure S6: Detection of *T. cruzi* DNA extracted with MagMAX Kit. *T. cruzi* parasites were spiked into human whole blood prior to extraction and then used at 120 (red), 12 (orange), 1.2 (yellow), 0.12 (green), 0.012 (blue), and 0 (black) parasite equivalents/reaction. Real-time curves show amplification at higher concentrations.

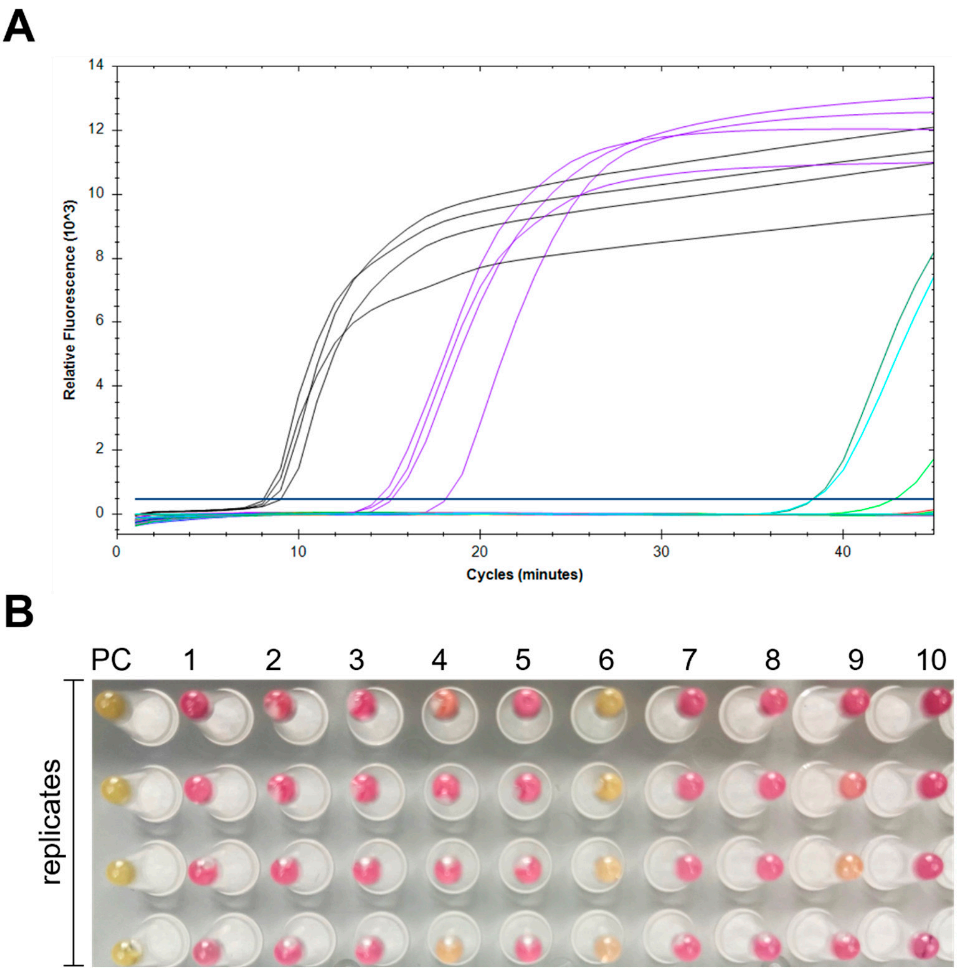

Figure S7: Identification of *T. cruzi* from canine blood samples – group 1. qPCR results were blinded from the researcher performing LAMP. (A) Real-time amplification curves and (B) end-point colorimetric results with 4 replicates at each concentration. Results represent 1 of 2 repeats. Samples were labeled as positive control (PC) (black), 1 (red), 2 (orange), 3 (yellow), 4 (green), 5 (blue), 6 (purple), 7 (pink), 8 (lime), 9 (turquoise), and 10 (lavender).

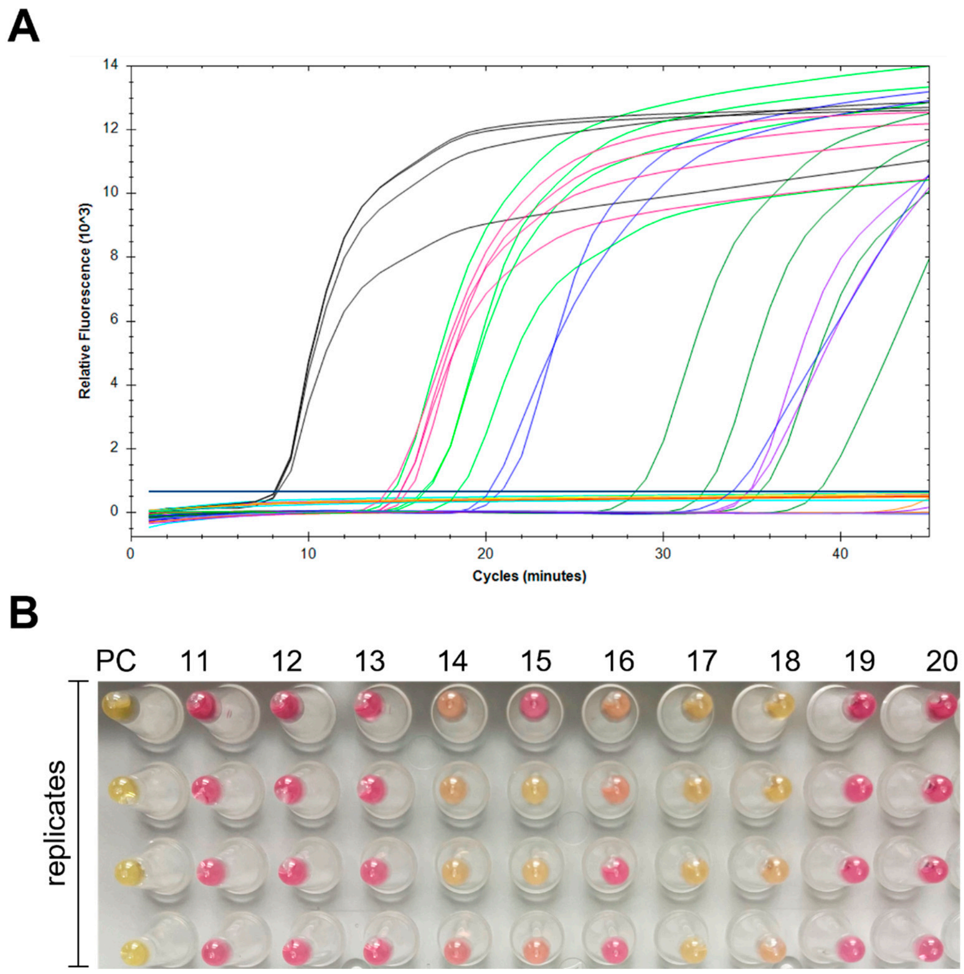

Figure S8: Identification of *T. cruzi* from canine blood samples – group 2. qPCR results were blinded from the researcher performing LAMP. (A) Real-time amplification curves and (B) end-point colorimetric results with 4 replicates at each concentration. Results represent 1 of 2 repeats. Samples were labeled as positive control (PC) (black), 11 (red), 12 (orange), 13 (yellow), 14 (green), 15 (blue), 16 (purple), 17 (pink), 18 (lime), 19 (turquoise), and 20 (lavender).
